# Early childhood caries and dental treatment need in low socio-economic communities in Cape Town, South Africa

**DOI:** 10.4102/hsag.v23i0.1039

**Published:** 2018-07-12

**Authors:** Nadia Mohamed, Jo M. Barnes

**Affiliations:** 1 Department of Paediatric Dentistry, Faculty of Dentistry, University of the Western Cape, South Africa; 2 Division of Community Health, Faculty of Medicine and Health Sciences, University of Stellenbosch, South Africa

## Abstract

**Background:**

Early childhood caries (ECC) is a particularly severe problem in low socio-economic communities which impacts the overall health and well-being of children. The extensive waiting lists for general anaesthesia and sedation services at the only tertiary dental care centre in the study area for the treatment of children with ECC were an indication of the extent of the problem. The true extent of the problem in this area was, however, not known. This information is crucial in order to plan and execute remedial measures.

**Aim:**

To assess the prevalence of oral and dental problems, especially ECC, in children under six years of age in the study population, and ascertain their need for dental treatment.

**Methods:**

A cross-sectional survey of 659 children from selected schools and clinics in the study area was carried out between 2010 and 2013.

**Results:**

A caries prevalence of 71.6% (472/659) was recorded. Of these, 67.5% (445/659) of children were in need of dental treatment.

**Conclusion:**

Over the last decade, there has been no improvement in the caries status of children in the study population, and no organised measures have been put in place to address this problem. Awareness needs to be raised so that governments, especially in developing countries, can take appropriate measures to alleviate this public health problem. Time and resources have to be invested in the education of all health professionals dealing with children, by raising their awareness of the early stages of the disease so that timeous referrals can be made.

## Introduction

Even though theoretically dental caries is a preventable disease (American Academy of Pediatric Dentistry [AAPD] 2017–2018), progress to reduce the prevalence of early childhood caries (ECC) has been slow. This is especially evident in the poorer, disadvantaged population groups in both developed and developing countries. These children remain particularly vulnerable to oral disease (Petersen et al. 2005). South Africa is not immune to this problem, and this was illustrated by a countrywide survey conducted between 1999 and 2000 (Van Wyk, Louw & du Plessis 2004).

Decay of the primary dentition is one of the main oral health reasons for which children are hospitalised (Plutzer & Spencer 2008). If left unchecked, severely decayed teeth can result in abscesses and cellulitis, which can progress to such an extent that it can, in rare cases, even be life-threatening (Begzati, Berisha & Meqa 2010). Caries can affect the feeding and overall well-being of the child. It can have impact on growth and cognitive development and on the child’s ability to concentrate and function optimally at school (Kumarihamy et al. 2011). The extent of this oral health problem is therefore of major significance as it undoubtedly affects the quality of life of many children, especially those who already live in suboptimal conditions (Petersen et al. 2005).

As children presenting with pain and infection are often under the age of five years, cooperation with dental treatment under local anaesthetic is usually poor. This means that more expensive treatment options such as general anaesthesia and sedation (which are associated with a greater risk of morbidity) have to be considered in most cases (Petersen et al. 2005; Tinanoff & Reisine 2009). This places a huge burden on existing resources (Kolisa, Ayo-Yusuf & Makobe 2013). As a large part of the budget has to be allocated to these specialised services, there is less funding available for prevention and basic restorative work.

Primary health care staff treat children for childhood illnesses from a young age. Yet, they are unaware that ECC could have impact on the child’s overall health and well-being. When compared with community health clinics in South Africa, in general, there are far fewer dental clinics. Children are therefore more likely to come into contact with primary health care staff during their formative years when they present for immunisations. Oral health is not a priority in many families, and parents often only take their children to the dentist when they experience pain. On the contrary, parents are more likely to seek advice from medical professionals for most health issues. Doctors and nurses are thus ideally placed to identify cases with the potential to progress to more life-threatening dental conditions like cellulitis. These health care workers should also be aware that dental caries share a common risk factor for other non-communicable diseases like obesity, diabetes, cardiovascular disease and cancer (Sheiham & Watt 2000).

The prevalence of ECC is influenced by a number of factors. Parental care-giving behaviour, socio-economic determinants, parental education and differences in lifestyle and culture are some of the important ones (Petersen et al. 2005). The AAPD defines ECC as:

the presence of one or more decayed (non-cavitated/cavitated lesions), missing (due to caries), or filled tooth surfaces in any primary tooth in a child 71 months of age or younger. (AAPD 2008)

Severe early childhood caries (s-ECC) refers to any carious lesion of the smooth surfaces, especially of the maxillary anterior teeth, in children younger than three years of age. This term describes a more rampant form of decay and is typically associated with infant feeding practices (Ismail & Sohn 1999). Nocturnal feeding and extended periods of bottle- and breastfeeding are detrimental to oral health (Bowen & Lawrence 2005; Prakash et al. 2012)

Throughout the literature, prevalence rates for ECC have varied considerably between different countries and population groups. Developing countries like India and Sri Lanka have reported prevalence figures of 36.42% and 32.19%, respectively (Kumarihamy et al. 2011; Tyagi 2009). It is important to note that ECC is predominantly a problem in the lower-income communities (Kopycka-Kedzierawski & Billings 2011; Kumarihamy et al. 2011; Petersen 2005; Tyagi 2009). Any health condition that affects large numbers of children and that needs scarce resources to treat is important, yet ECC has not received the attention commensurate with its impact on the community and health systems. This is highlighted by the large numbers of children in need of dental treatment under general anaesthesia (Mohamed & Barnes 2008; Peerbhay & Barrie 2012).

The only reliable and fairly recent determination of ECC in South Africa was a National Children’s Oral Health Survey conducted in South Africa between 1999 and 2000 and included children between the ages of 4 and 15 years (Van Wyk et al. 2004). The Western Cape (the province in which this study was conducted) was shown to have the highest caries prevalence across all age groups. The prevalence of dental caries in 4- to 5-year-old children in the Western Cape was recorded at 77.1% (Van Wyk et al. 2004). No data were available for children younger than 4 years of age. This survey, which was conducted more than 15 years ago, included children of all socio-economic groups, thus including subgroups with a much lower prevalence of ECC. No new comprehensive data on this condition have been gathered, and all planning is still based on this survey.

A true prevalence study of ECC in any area requires both the ascertainment of all the cases of ECC and a detailed database of the population in whichever age group is being studied in the same area. Accurate information of this nature is not available at community level for many districts in South Africa. To complicate matters, the accuracy of the available official South African census data has been questioned (Paton 2012).

The Tygerberg Oral Health Centre (TOHC) is the only public oral health hospital in the entire metropolitan area of the City of Cape Town. The growing waiting lists for sedation and general anaesthesia to treat the consequences of ECC in children indicated that the high caries prevalence had reached significant proportions in this sector of the population. A retrospective, records-based study provided a sense of the extent of ECC in this area as many of these children were treated under general anaesthesia (Mohamed & Barnes 2008). The results of this 2008 study were used to refine the data collection tools used in the present study. However, only children presenting at the TOHC for treatment were included in this retrospective study, while the number of children in the community at large who were unable to access treatment was unknown. The TOHC-based observations therefore needed confirmation in a community-based survey that included children who were not sampled on any oral health care service premises.

The purpose of this study is thus to assess the prevalence of oral and dental problems, especially ECC, in children under six years of age in the study population and to ascertain their need for dental treatment.

## Methods

A cross-sectional, oral health survey of children from various lower socio-economic areas draining to the TOHC was conducted.

### Ethics approval

All consent forms were separated from data capture sheets so as to preserve patient anonymity. There were no forms of identification on the actual data capture sheets. Parents or caregivers placed the signed consent forms into a sealed box (with a slot cut out in the lid) themselves. This box was kept in the corner of the waiting room, separated from another sealed box into which the completed data capture sheets were deposited by the researcher. Patient anonymity was thus preserved as the consent forms could not be matched to the data capture sheets. Consent was obtained on the day of the examination, and the data capture sheets were completed at the same visit. The boxes were transported from site to site and were only opened after all the data had been collected.

### Study population

The municipal officials in charge of the indigent policy applications in this part of the City of Cape Town were approached for identifying the areas where the largest number of persons did not pay any municipal rates and taxes or were receiving the indigent benefits made available by the City. Health and preschool facilities situated in these low-income suburbs draining to the TOHC were identified. All facilities where the necessary permission could be obtained were included (4 preschools and 11 community health clinics).

The children selected to participate at the clinic sites were only those who accompanied other persons (mainly their caregivers) so that they were deemed to be ‘healthy’ (no obvious health complaints) at the time of the study. In order to lessen the impact of possible selection bias (i.e. only children associated with health care institutions), the sample was extended to include children from the government-funded daycare centres and crèches in the area.

### Inclusion criteria

All children older than six months and below six years of age who were present at the above-mentioned facilities on the day of the research visit, but did not present with any health complaints themselves and whose caregivers gave consent, were selected for the survey.

### Exclusion criteria

- Children presenting with a medical condition that could have a direct impact on oral health status were excluded.
- Children younger than six months without any erupted teeth were also excluded.

### Data collection

Data (i.e. dental status and treatment need) were recorded on data capture sheets and entered into a Microsoft Excel spreadsheet.

### Preschools or crèches

Four preschools or crèches were included in the survey. Consent forms were given to the children to take home for their parents to sign and were returned to the school. Children who did not have valid consent forms on the day of the research visit were excluded from the study. After the examination was conducted, each child received a written note to take home informing their parents of the results of the examination. If further treatment was needed, contact details of the nearest facility where treatment could be sought, were provided.

### Community and well-baby clinics

Eleven community health and well-baby clinics were included in the study. Parents gave consent for their children to be subjected to an oral examination.

### Sample size

No formal calculations of minimum sample sizes were carried out, as is the case for inferential statistics where the usual requirements of sufficient accuracy and statistical power are demanded. This could be seen as a limitation of the study. In keeping with the approach utilised in survey methodology, the sample sites were selected to provide as representative a group of participants (children) as possible.

A total of 700 children were examined. Of these, 41 were 6 years of age and were therefore not included in the study database as permanent teeth erupt at the age of 6 years. To keep charting consistent, the study was confined to examining the primary dentition only. A total of 659 children between the ages of 6 months and just below 6 years comprised the final sample.

### Method of examination

Oral examinations took place between 2010 and 2013 and were solely conducted by the author who is a paediatric dentist with 17 years of experience. This ensured consistency in caries diagnosis. The intention was merely to record the presence of caries and thus assess the treatment need. The extent of the lesion and degree of cavitation of each individual tooth was therefore not recorded.

Caries diagnosis was made on an entirely visual basis using modified ICDAS and ICDAS-LAA criteria for visual detection of caries (ICDAS II Criteria Manual 2009). The ICDAS-LAA criteria (ICDAS II Criteria Manual 2009) had specific, detailed descriptions of ‘active’ lesions which were followed to the letter.

Missing (because of extraction) and filled teeth were also recorded as these variables indicate prior dental treatment. The presence of root rests, abscesses and soft tissue lesions was also noted together with reports of pain and the need for orthodontic treatment.

The definitions of ECC and s-ECC as proposed by the AAPD were used in this study (AAPD 2008). The position of the caries distinguishes the one caries distribution pattern from the other where s-ECC characteristically involves the maxillary anterior teeth. Caries of posterior teeth develops later and can be because of factors other than infant feeding practices (Sayegh et al. 2002).

The study design was cross-sectional, and there were no repeat measurements. There were also no measurements by more than one assessor. Therefore, the traditional tests of inter- and/or intra-reliability of measurements such as Cronbach’s alpha or the Kappa statistic were not applicable to the data set. The assessments needed for scoring by means of the ICDAS criteria are very uncomplicated by design. Although the single assessor design greatly contributes to repeatability of assessments, it does not entirely eliminate the chance of bias in assessments. The ICDAS criteria are, however, simple and very explicit and need only basic skills that every clinical dentist should be able to carry out with minimal variation. The chance of bias in recording the data should be seen as a possible limitation of the study as questionnaires were completed by a single researcher.

### Ethical considerations

This study was approved by the Research and Ethics Committee of the Faculty of Health Sciences of the University of Stellenbosch (Registration number NO7/10/225). Consent was obtained from the Provincial Department of Health and City Health (City of Cape Town) to conduct the study at various community clinics. Once provincial approval was obtained, the superintendent of each community health facility and the principal of each school where children were examined were then approached for permission to conduct the study. Written informed consent was obtained from the parents of the children who were examined.

## Results

Of the 659 children in the study, 357 were males (54.2%) and 302 (45.8%) were females.

### Overall caries distribution pattern

Table 1 shows the overall caries distribution pattern.

**TABLE 1:** Overall caries distribution pattern.

Caries distribution pattern	Number (*n* = 659)	Percentage of total
s-ECC	149	22.6
ECC	62	9.4
s-ECC and ECC	261	39.6
Total number affected by caries, past or present	472	71.6
Caries free	187	28.4

ECC, early childhood caries; s-ECC, severe early childhood caries.

### Active caries distribution pattern per age group

Table 2 shows the active caries distribution pattern per age group.

**TABLE 2:** Active caries distribution pattern per age group.

Active caries per age group	Percentage of active caries (*n* = 445)		
	s-ECC only % (*n* = 131)	ECC only % (*n* = 58)	s-ECC and ECC combined % (*n* = 256)
1 year to 1 year 11 months (*n* = 50)	82 (41)	6 (3)	12 (6)
2 years to 2 years 11 months (*n* = 81)	50.6 (41)	6.2 (5)	43.2 (35)
3 years to 3 years 11 months (*n* = 128)	25.8 (33)	13.3 (17)	60.9 (78)
4 years to 4 years 11 months (*n* = 105)	11.4 (12)	18.1 (19)	70.5 (74)
5 years to 5 years 11 months (*n* = 81)	4.9 (4)	17.3 (14)	77.8 (63)

Note: Actual numbers of children affected are reflected in brackets.

ECC, early childhood caries; s-ECC, severe early childhood caries.

### Overall caries distribution pattern per age group (past and present caries experience)

Table 3 shows the overall caries distribution pattern per age group (past and present caries experience).

**TABLE 3:** Overall caries distribution pattern per age group (past and present caries experience).

Age distribution	No. of children	Percentage of caries free per age group (*n*)	Total no. of children with caries per age group (%)	Percentage with ECC per age group (*n*)	Percentage with s-ECC per age group (*n*)	Percentage with ECC and s-ECC per age group (*n*)
6 to 11 months	10	100	0	0	0	0
1 year to 1 year 11 months	103	49.5 (51)	52 (50.5)	3 (3)	41.7 (43)	5.8 (6)
2 years to 2 years 11 months	129	32.6 (42)	87 (67.4)	3.9 (5)	36.4 (47)	27.1 (35)
3 years to 3 years 11 months	172	22.1 (38)	134 (77.9)	9.9 (17)	21.5 (37)	46.5 (80)
4 years to 4 years 11 months	142	20.4 (29)	113 (79.6)	14.1 (20)	12 (17)	53.5 (76)
5 years to 5 years 11 months	103	16.5 (17)	86 (83.5)	16.5 (17)	4.9 (5)	62.1 (64)

*Note*: Actual numbers of children affected are reflected in brackets.

ECC, early childhood caries; s-ECC, severe early childhood caries.

### Previous dental treatment

Of the children who had previously visited a dentist, only three children had received restorative treatment. One hundred out of 107 children (93%) received extractions.

### Presence of pain

The vast majority of children (97.6%) did not present with pain.

### Data analysis

The data were transferred by a statistician at the Centre for Statistical Analysis at the University of Stellenbosch into Statistica version 11 (StatSoft Inc. 2012, USA) for further analysis. Chi-squared analysis was carried out. Data capture was monitored by taking random samples of records and checking the data entry. Data integrity was monitored by the study supervisors and the statistician during the analysis and reporting of the data.

## Discussion

This study, which was conducted in various lower socio-economic areas draining to the TOHC, found that a disquieting 71.6% of children aged > 6 months to < 6 years of age were affected by caries (Table 1).

Unlike the National Children’s Oral Health Survey (Van Wyk et al. 2004) which included the entire Western Cape, this study only included the low-income areas of the City draining to the TOHC. The NCOHS revealed that caries prevalence in the Western Cape in the 4- to 5-year-old age group was estimated to be 77.1% at the time (Van Wyk et al. 2004). This is far higher than the projected goal of 50% caries-free children set by the Department of Health for 2000 (Van Wyk et al. 2004). More than a decade later, this goal has still not been achieved. In fact, there has not been any improvement, as in this study, of the 245 children examined in the 4- to 5-year-old category, 186 (75.9%) presented with active caries which required intervention (Table 2). The actual caries prevalence in this age group is reflected by the total number of children that were affected by caries at some stage, past or present and comprised 199 (81.2%) children (i.e. 199/245) (Table 3).

In this study, s-ECC was noted in 62.2% of the children examined (Table 1). Children as young as 1 year of age were affected by caries. Exclusive s-ECC was most prevalent in the 1-year to 1-year-11-month age category with 41.7% of children affected (Table 3). Implementing prevention at the time of tooth eruption or earlier would therefore be most beneficial.

In this study, 62.8% of the 2-year-old children and 77.9% of 3-year-olds presented with active caries. The prevalence peaked at 3 years of age, where 74.4% (i.e. 128/172) of children in this age category presented with active caries (Table 2). In contrast, approximately 25% of 3-year-olds in Mpumalanga were reported to have caries in 2006, which is lower than studies reported from other parts of the world (Wanjau 2006). This highlights the fact that younger children should be targeted during preventative campaigns, especially those in the vulnerable two- to three-year age group. Early intervention introduced before the age of three years is therefore crucial to help curtail caries development in this vulnerable group.

Changes in surface structure, that is, demineralisation (which indicates the initial stages of the disease process) was noted in 36 children. These lesions have the potential to progress if left unchecked.

Health care staff can play an important role in early identification of these initial lesions and timeous referral of these patients for preventive measures which can arrest the caries process. The AAPD advises that all health care professionals be empowered regarding the aetiology and prevention of ECC. They should be able to give basic advice regarding feeding practices and diet and encourage parents to take their children for their first dental visit by the age of 12 months. The Road to Health booklet of the Department of Health already makes provision for dental visits. The importance of regular dental attendance should therefore be emphasised so that problems can be identified early and more severe complications can be avoided.

Reinforcing information regarding good feeding and oral hygiene practices can go a long way towards helping to reduce the caries prevalence. The use of nursing bottles, sippy cups and sports drink bottles with sip-friendly caps should be discouraged, especially at bedtime. Parents should also assist children to brush their teeth until the age of approximately 10 years as they do not have the necessary dexterity to perform this task adequately before this age (Sandström, Cressey & Stecksén-Blicks 2011). Oral hygiene practices should be implemented at least once a day and especially last thing at night. The importance of preserving the primary dentition should also be emphasised so as to prevent malocclusions and the need for possible future orthodontic treatment. In a study conducted in Mpumalanga Province in 2006, most parents opted to have their children’s teeth extracted (Ferreira et al. 2007). The belief that ‘primary teeth will be replaced’ is one of the main reasons why the primary dentition is neglected and treatment is not sought by parents (Begzati et al. 2010).

### Treatment need

The majority of the children in this study, that is, 67.5% (i.e. 445/659) had evidence of carious activity which needed some form of dental treatment. The unmet treatment need is calculated by ‘dividing the percentage of untreated caries by the caries prevalence’ (Van Wyk & Van Wyk 2004). Using this formula, the unmet treatment need of 4- to 5-year-old children in the Western Cape in the 2004 survey was calculated to be 93.3% compared with 94.3% of this study (i.e. 67.5/71.6 or untreated caries/caries prevalence). This shows that, to date, this problem has not been addressed at all. This situation occurs in other parts of South Africa as well. Nearly all 3- to 5-year-old preschool children examined in Mpumalanga’s Philadelphia district in 2006 were in need of dental treatment with only 0.7% of affected teeth reportedly treated (Wanjau 2006).

### Types of dental treatment reported

Of the children surveyed in this study, only 16.2% had been to a dentist before. Extractions were the treatment of choice in 95% of cases (102/107). It is a source of concern that 40 children under the age of four years received extractions. This is because of the severity of the condition early in life and highlights the need to target this group of children in particular for intervention programmes.

Only three children had restorations placed. The numbers of restored teeth recorded in all age groups in the 2004 survey were also reported to be ‘negligible’ (Van Wyk & Van Wyk 2004).

Generally, in South Africa, the need for preventive and restorative treatment is greater than for extractions (Peerbhay & Barrie 2012; Van Wyk & Van Wyk 2004). The number of children needing treatment indicates that, because of a lack of resources, there are not enough dental services which target school children (Van Wyk & Van Wyk 2010; Wanjau 2006).

By introducing a preventive programme early in life, the need for restorative treatment can be delayed until children are older and display better cooperation (Pienihäkkinen, Jokela & Alanen 2005). This would significantly reduce the demand for general anaesthesia and sedation services and thereby cut costs, especially if one takes into consideration that the health care system is already overburdened.

### Presence of pain

Despite the high caries prevalence, only 16 out of the 659 children (2.4%) in this survey claimed to experience pain. This was as a result of severe infections resulting from carious lesions that had progressed quite far. Of these, seven children fell into the four-year age group.

Because of its transient nature, the degree of dental pain is difficult to assess, and this could also contribute to the fact that it often goes unreported (Low, Tan & Schwartz 1999). As the presence of pain is usually relayed by the parents, especially in children, the actual number experiencing pain could be higher (Bastos et al. 2008).

## The current situation

The World Health Organization’s goals for various oral health indicators, including ECC, stated that by 2010, 90% of five-year-old children should be free of caries (Hobdell et al. 2000). At the present prevalence of 71.6% in children younger than six years of age found in this study, it is clear that this goal has not been reached by any means. It also underlines the lack of attention to this problem.

A Comprehensive Oral Health Service Plan (COHSP 2010) based on the South African National Oral Health Strategy was compiled in a bid to revamp the oral health services so as to meet the health care goals for 2010. Even though this proposal was signed by the Minister of Health in 2007, it has not been formally accepted or published. It was intended to be used as a guide for allocating resources and implementing oral health programmes.

Addressing ECC and managing caries in the primary and permanent dentitions were identified as primary concerns. Oral health promotion targeting mothers and children, brushing and rinsing programmes at schools and placement of fissure sealants were suggested as a means to address the problem of dental caries. These suggestions were based on data obtained from National Oral Health surveys in 1988 1989 and 2002 (Van Wyk & Van Wyk 2004). This showed that over the last 20 years not much progress has been made and that none of the national goals for 2010 have been met.

The current focus of the proposed service plan is on public health service efforts regarding the permanent dentition. It would, however, make more sense to invest in the preservation of the primary dentition as early extractions lead to malocclusions. Investing in prevention and the preservation of the primary teeth would therefore make more sound economic sense.

With the present huge demand for general anaesthesia services (Peerbhay & Barrie 2012) and the low number community dental clinics that are also ill-equipped and unable to provide basic restorative care, the waiting lists at tertiary institutions like the TOHC are overflowing and cannot cope with this demand. There is therefore a need to increase the number of facilities that are able to provide this service, especially in the rural areas.

As there is a greater need for restorative services, more state funds need to be invested in upgrading facilities to cope with the unmet treatment need so that the backlog can be addressed. At present, community dental clinics almost exclusively provide only an emergency extraction service. By improving the services that primary health care clinics are able to provide such as the inclusion of preventive and restorative services, the numbers of referrals to tertiary institutions would be reduced and waiting lists would be alleviated. In addition to upgrading the services, awareness must be raised regarding the causes of the disease and how to prevent it. This would help to reduce the number of new cases and prevent the recurrence of caries in those individuals who have already been affected. The role of medical and allied health professionals in this process is therefore crucial.

## Conclusion

The current situation is a red flag which highlights the need for a new approach to prevent caries by targeting pregnant mothers and younger children in order to address this public health problem that has reached epidemic proportions in the Western Cape. This can be achieved through collaboration of health care professionals from all sectors.

Petersen et al. (2005) showed that the cost of managing dental caries has a significant economic impact. It is the fourth most expensive disease to treat. Despite the fact that ECC is largely preventable, hundreds of thousands of rands are spent each year on anaesthetist fees and theatre costs. With a shrinking health budget and the arrival of infectious diseases of epidemic proportions such as HIV and AIDS, the prevention of ECC and its consequent cost savings can be an extremely positive investment of health funds. Oral health matters should therefore be prioritised and incorporated into national health programmes and in so doing improve general health and quality of life of the community at large. By emphasising prevention and by reducing the prevalence of ECC, the need for more expensive treatment options such as general anaesthesia and sedation services can be alleviated.
